# Organotypic Tissue Culture of Adult Rodent Retina Followed by Particle-Mediated Acute Gene Transfer In Vitro

**DOI:** 10.1371/journal.pone.0012917

**Published:** 2010-09-23

**Authors:** Satoru Moritoh, Kenji F. Tanaka, Hiroshi Jouhou, Kazuhiro Ikenaka, Amane Koizumi

**Affiliations:** 1 Division of Correlative Physiology, National Institute for Physiological Sciences, Okazaki, Japan; 2 Division of Neurobiology and Bioinformatics, National Institute for Physiological Sciences, Okazaki, Japan; 3 Section of Communications and Public Liaison, National Institute for Physiological Sciences, Okazaki, Japan; 4 Department of Physiological Sciences, School of Life Science, The Graduate University for Advanced Studies (SOKENDAI), Okazaki, Japan; 5 Drug Safety Research Laboratories, Astellas Pharma Inc., Osaka, Japan; Oregon Health & Science University, United States of America

## Abstract

**Background:**

Organotypic tissue culture of adult rodent retina with an acute gene transfer that enables the efficient introduction of variable transgenes would greatly facilitate studies into retinas of adult rodents as animal models. However, it has been a difficult challenge to culture adult rodent retina. The purpose of this present study was to develop organotypic tissue culture of adult rodent retina followed by particle-mediated acute gene transfer in vitro.

**Methodology/Principal Findings:**

We established an interphase organotypic tissue culture for adult rat retinas (>P35 of age) which was optimized from that used for adult rabbit retinas. We implemented three optimizations: a greater volume of Ames' medium (>26 mL) per retina, a higher speed (constant 55 rpm) of agitation by rotary shaker, and a greater concentration (10%) of horse serum in the medium. We also successfully applied this method to adult mouse retina (>P35 of age). The organotypic tissue culture allowed us to keep adult rodent retina morphologically and structurally intact for at least 4 days. However, mouse retinas showed less viability after 4-day culture. Electrophysiologically, ganglion cells in cultured rat retina were able to generate action potentials, but exhibited less reliable light responses. After transfection of EGFP plasmids by particle-mediated acute gene transfer, we observed EGFP-expressing retinal ganglion cells as early as 1 day of culture. We also introduced polarized-targeting fusion proteins such as PSD95-GFP and melanopsin-EYFP (hOPN4-EYFP) into rat retinal ganglion cells. These fusion proteins were successfully transferred into appropriate locations on individual retinal neurons.

**Conclusions/Significance:**

This organotypic culture method is largely applicable to rat retinas, but it can be also applied to mouse retinas with a caveat regarding cell viability. This method is quite flexible for use in acute gene transfection in adult rodent retina, replacing molecular biological bioassays that used to be conducted in isolated cultured cells.

## Introduction

Organotypic tissue culture techniques of adult rabbit retinas in vitro established by Koizumi et al [1], [2] have been applied widely to other retinal neurophysiological research with variable gene transfer [3], [4]. However, because of limited rabbit genome resources, application of this organotypic culture for further variable gene manipulation has been restricted. In addition, rodents such as mice and rats became more important as animal models for retinal and ophthalmological research (for example, transgenic mouse lines in [5]). Therefore, a tissue culture system using the adult rodent retina in combination with further gene manipulation would be required for the study of retinal neurophysiology and ophthalmological research.

The differences between the rabbit and rodent retina are thickness and intra-retinal vascularity; the rodent retina is thicker and vascularized [6]. Survival of adult rodent retina depends on intra-retinal vascular circulation, in contrast to that of adult rabbit retina which is so thin that survival is mainly dependent on diffusion. Thus, the in vitro tissue culture of the adult rodent retina has been a difficult challenge.

Here we report a new whole-tissue adult rodent retinal culture system with acute gene transfer using particle-mediated transfer (i.e. the gene-gun) in vitro. Our primary goal was to optimize the culture protocol for the adult rodent retina. We also showed that our organotypic tissue culture with an acute gene transfer system successfully enabled the efficient introduction of transgenes into neurons of the adult rodent retina.

## Results

### Interphase culture system for organotypic tissue culture of the adult rodent retina

**Figure 1:**
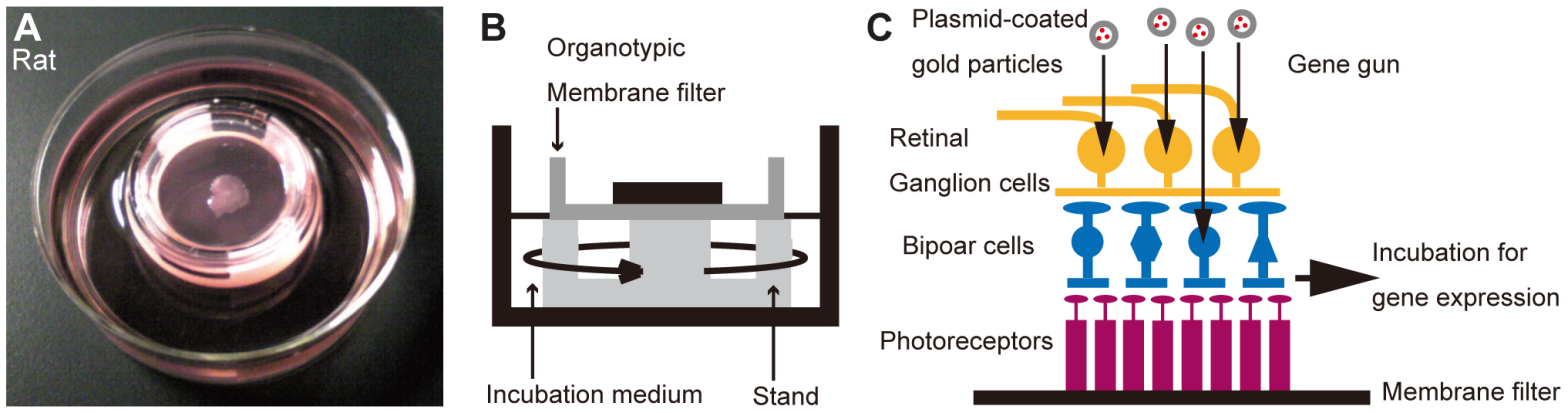
Organotypic tissue culture of adult rodent retina and particle-mediated acute gene transfer. Photograph (A) and schematic diagram (B) of the interphase chamber. Deep dishes were used with custom-made stands to support the tissue culture insert with the flat-mounted retina. The retina was in contact with the medium over the filter on the photoreceptor side, the ganglion cell side faced the atmosphere. (C) Schematic diagram of particle-mediated transfer of plasmids into the rodent retina.

Adult rodent retinal culture (Figures 1A and 1B) was performed with modifications to the published protocol of interphase culture system of the adult rabbit retina [1], [2]. We implemented three optimizations from previous interphase culture system for adult rabbit retinas: First, during culture, we added a greater volume (>26 mL) of Ames' medium per retina. Second, we agitated the medium at a higher speed (constant 55 rpm) by rotary shaker in a CO_2_ incubator at 37°C. Third, we supplemented the medium with a greater concentration (10%) of horse serum. Particle-mediated acute gene transfer (gene-gun) was conducted from the retinal ganglion cell side (Figure 1C).

### Sustained retinal structural organization after four days of culture

To confirm that the adult rodent retina (>P35 of age) were structurally intact after 4 days of culture, we examined the vertical organization of retinal layers and the horizontal whole-mount organization of the cultured specimens.

**Figure 2:**
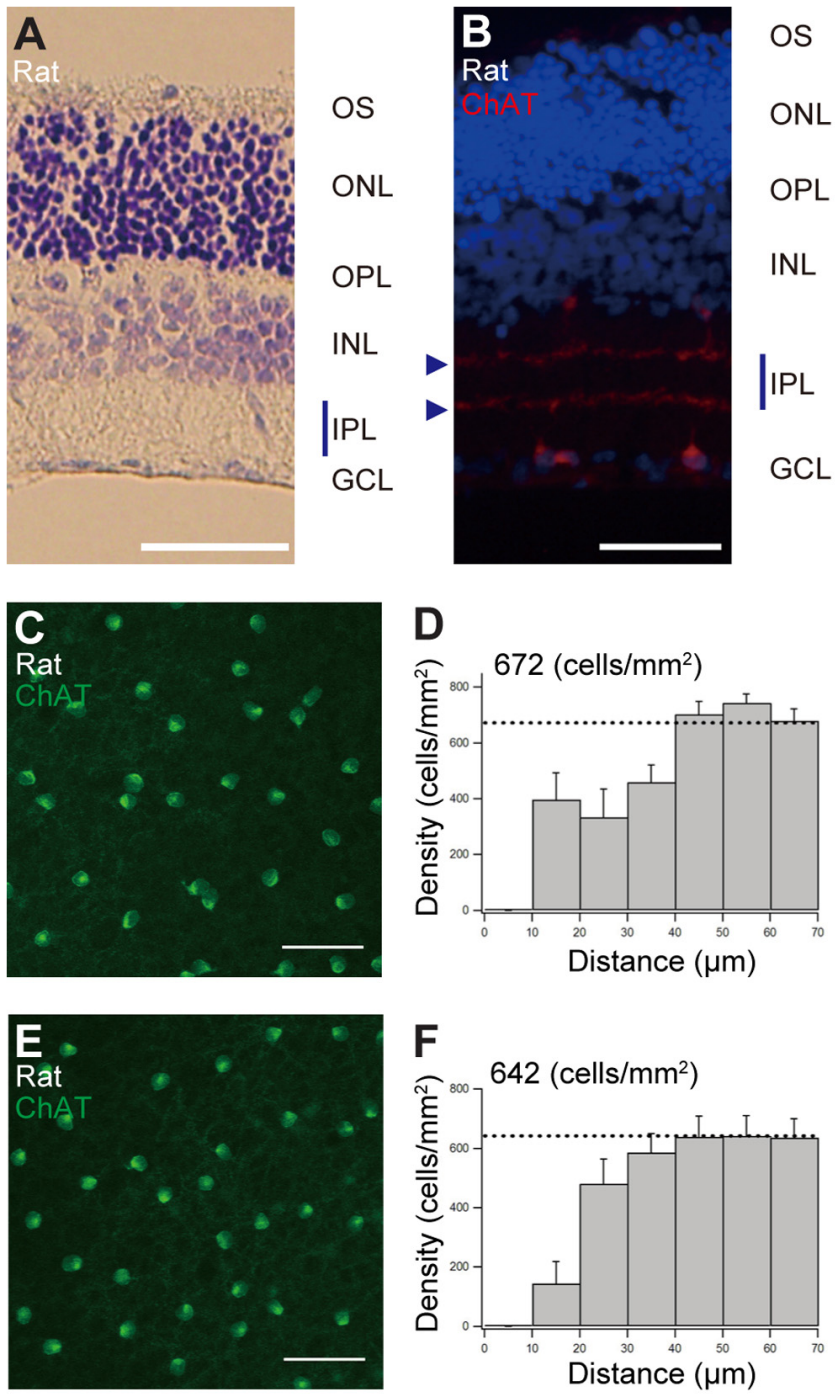
Vertical and whole-mount horizontal structures of adult rat retina after 4 days of culture. (A and B) Vertical organization of retinal layers after 4 days of culture. (A) Nissl staining of vertical sections of rat retina after culture. (B) Vertical sections were stained with an antibody against ChAT (red) and Hoechst 33342 (blue) after 4 days of culture. ChAT-positive cells are starburst amacrine cells. GCL, ganglion cell layer; INL, inner nuclear layer; IPL, inner plexiform layer; ONL, outer nuclear layer; OPL, outer plexiform layer; OS, photoreceptor outer segments. Scale bars for (A) and (B), 50 µm. (C–F) Horizontal organization of whole-mount retina after 4 days of culture. (C and E) Typical whole-mount images of rat retinal starburst amacrine cells stained with an antibody against ChAT (green) after 4 days of culture. (C) Field with focus on the GCL (ON starburst amacrine cells). (E) Field with focus on the INL (OFF starburst amacrine cells). These starburst amacrine cells after culture showed typical regular mosaic distribution. Scale bar for (C) and (E), 50 µm. (D and F) Density recovery profile for starburst amacrine cells in the GCL (D) and in the INL (F).

In vertical slices after 4 days in culture, the retinal layers were structurally intact. Figure 2A shows Nissl staining of vertical slices of adult rat retina after 4 days in culture. Each retinal layer was clearly identified; in the inner plexiform layer, there were very clear double bands of starburst amacrine cells' dendritic layers (Figure 2B, arrowheads). These results indicate that the vertical structures of the retinal layers were structurally intact.

In the horizontal whole-mount retina after 4 days in culture, the typical regular mosaic organization of starburst amacrine cells in the ON and OFF layers were observed (Figures 2C and 2E). Statistical DRP analysis revealed that starburst amacrine cells were regularly organized after 4 days in culture. The regular spacing of 40 to 50 µm was well maintained (Figures 2D and 2F). The distribution of starburst amacrine cells in whole-mount mice retina after 4 days in culture showed regular mosaics (data not shown); these features were quite similar to those shown in previous reports of in vivo studies [7].

The analysis of vertical and horizontal whole-mount structures of adult rodent retina after 4 days in culture provided clear evidence that retinal structures were well maintained in vitro using our interphase culture system.

### Cell viability assay for cultured retinas

**Figure 3:**
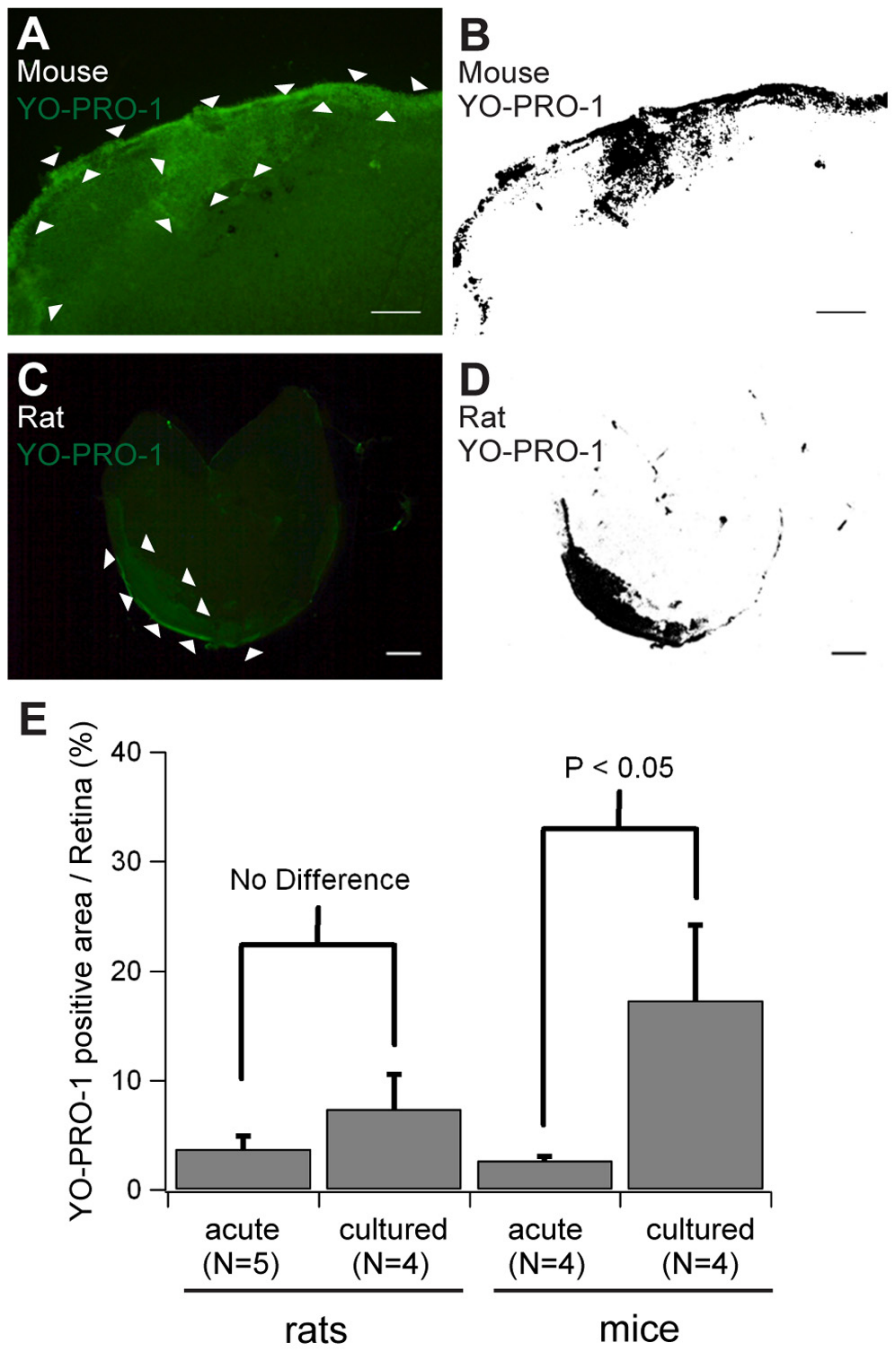
Cell viability assay on cultured retinas of rats and mice. Mouse retina (A and B) and rat retina (C and D) after 4-day culture were stained with YO-PRO-1 (undergoing apoptotic cell marker). YO-PRO-1 positive cells were clustered along with the edge of cultured retinas of mice and rats (surrounded by arrow heads in A and C). The clusters formed area with bright fluorescence so that we defined a clustered area as a YO-PRO-1 positive area (black areas in B and D). Note: panels (C and D) show most severely damaged rat retina after 4-day culture (12.0% area was positive for YO-PRO-1). Scale bars for (A and B), 500 µm. Scale bars for (C and D), 1 mm. (E) Quantification of YO-PRO-1 positive area on rat and mouse retinas. In the rat retinas, 7.4±3.2% (mean ± standard deviation, n = 4) of the whole retinal area was YO-PRO-1 positive, while 3.7±1.2% (n = 5) of retinal area were also YO-PRO-1 positive even in acutely isolated retina (no significant difference). In the mouse retinas, 17.3±6.9% (n = 4) of the whole retinal area was YO-PRO-1 positive, significantly larger than that of acutely isolated mouse retinas (2.7±0.4%, n = 4). Significant difference was detected in mouse retinas by Student's t-test.

To evaluate retinal cell viability after culture, we labeled cells undergoing apoptosis (and dead cells) with YO-PRO-1 after 4-day culture of rat and mouse retinas [1]. Cells undergoing apoptosis and dead cells were supposed to show green fluorescence; live cells were to show little or no fluorescence. We found that YO-PRO-1 positive cells were clustered along the edge of cultured retinas and formed a distinct area with bright green fluorescence (Figures 3A and 3C). We defined a bright green area as a YO-PRO-1 positive area (Figures 3B and 3D), and quantified YO-PRO-1 positive areas on rat and mouse retinas, comparing with those on acutely isolated retinas. In the rat retinas, 7.4±3.2% (mean ± standard deviation, n = 4) of the whole retinal area was YO-PRO-1 positive (Figure 3E), while 3.7±1.2% (n = 5) of the retinal area was also YO-PRO-1 positive in the acutely isolated retina (no significant difference). In the mouse retinas, 17.3±6.9% (n = 4) of whole retinal area was YO-PRO-1 positive, significantly more than seen with acutely isolated mouse retinas (2.7±0.4%, n = 4; Figure 3E). Except for these concentrated YO-PRO-1 positive areas, YO-PRO-1 positive cells were sparsely located with a density of less than 0.5% of total cells, similar to that seen with cultured rabbit retina [1]. Cultured mouse retinas exhibited less viability, but more than 80% of the remaining retinal area was still viable.

### Electrophysiology of cultured retinas

**Figure 4:**
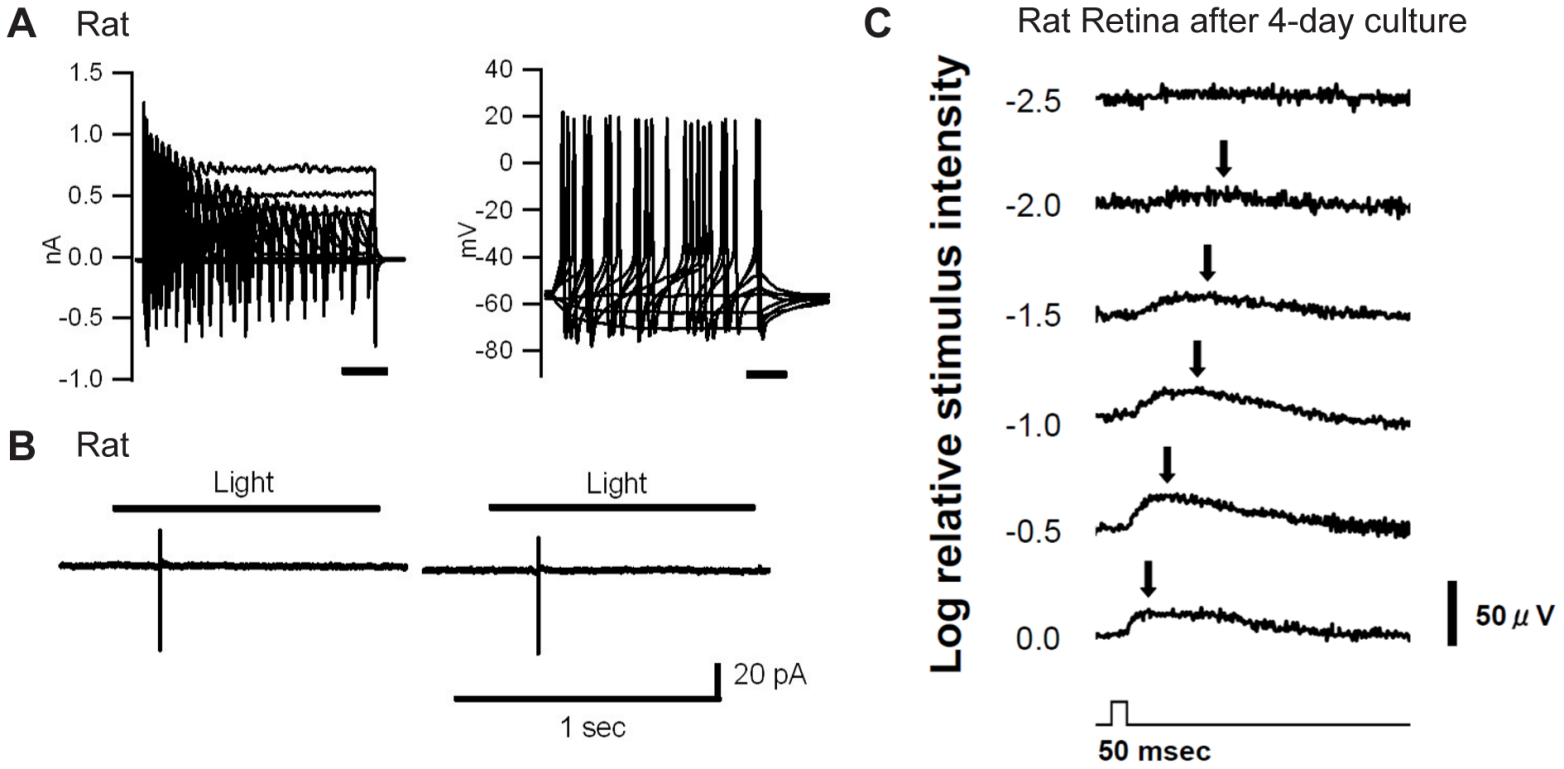
Electrophysiological recordings from cultured rat retinas. (A) Whole-cell patch-clamp recordings of a rat retinal ganglion cell after 4-day culture under voltage clamp and current clamp configurations. Left: Voltage-clamp recordings. Holding voltage  = −71 mV. Voltages were clamped from−91 mV to +19 mV, in 10-mV steps, for 100 msec. Right: Current-clamp experiment. Injected currents were from −20 pA to 50 pA, in 10-pA steps, for 100 msec. Resting membrane potential was −57 mV. This cell did not show any light responses. (B) Loose cell-attached patch-clamp recordings of a rat retinal ganglion cell under voltage-clamp cell-attached configuration after 4-day culture. A pipette voltage was clamped at 0 mV. One-second exposures to whole-field illumination were applied at 1200 cd/m^2^. Cells in (A) and (B) were different. (C) Trans-retinal field potential recording elicited by white LED light stimulus of a rat retina after 4-day culture. This field potential recording was an imitation of in vitro electroretinogram**s** (see Figure S1). The light-induced positive potentials were observed in proportion to the intensity of light (arrows). Maximum luminance was 450 cd/m^2^ at the surface of retina.

To demonstrate neurophysiological applicability of the preparation, we conducted electrophysiological evaluations of cultured rat retinas. Patch-clamp recordings showed that all rat retinal ganglion cells after 4-day culture (n = 10 cells) were able to generate action potentials spontaneously or by current injection (Figure 4A). However, light responses became less reliable; four out of ten cells showed action potentials responding to exposure of light stimuli (Figure 4B), but light responses were not tolerated with repetitive light exposure. For further evaluation, we examined light responses using trans-retinal field potential recordings, which were an imitation of electroretinograms in vitro [8] (see Figure S1). The electrical responses of trans-retinal field potential recordings were supposed to represent the sum of all electrical activity of the retinal neurons following exposure to a light stimulus. After 4 days, four out of nine retinas showed light responses in proportion to light intensity, but the amplitudes of light responses were smaller than those seen with acutely isolated retinas (Figure 4C in comparison with Figure S1B). In particular, positive potentials were detected clearly in proportion to light intensity (arrows in Figure 4C), but other types of light-induced potentials such as the negative potentials observed in acutely isolated retinas (Figure S1) were not detectable. Even at 2 days of culture, these light responses were quite weak (data not shown). These weaker light responses might be related to shorter outer segments of photoreceptors after culture following the isolation from retinal pigmental epithelium. In summary, individual retinal ganglion cells in cultured rat retinas were still physiologically viable to generate action potentials, but cells exhibited weak or no light responses probably due to loss of pigmental epithelium and shorter outer segments of photoreceptors.

### EGFP expression in adult rodent retina as early as one day in culture

**Figure 5:**
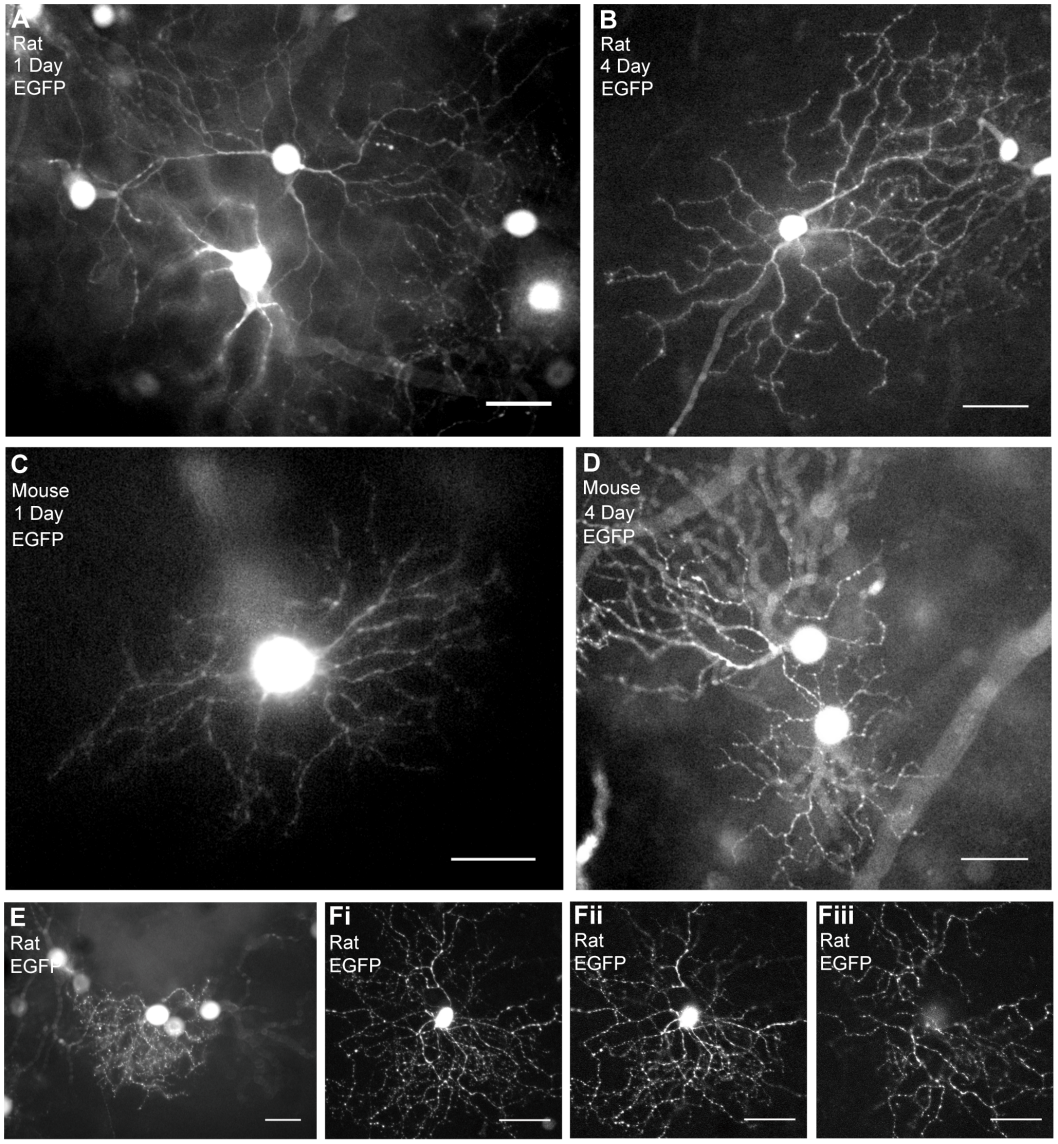
EGFP expression in rat and mouse retinal ganglion cells after culture. (A) EGFP expression of transfected rat retina after 1 day in culture. We observed EGFP in the entire dendritic arbor with fine dendrites. (B) EGFP expression of transfected rat retina after 4 days in culture. (C) EGFP-expressing retinal ganglion cells in mouse retina after 1 day in culture. (D) EGFP expression of transfected mouse retina after 4 days in culture. (E and F) EGFP expression in different subtypes of retinal ganglion cells. (E) EGFP expression in a rat retinal ganglion cell with monostratified busy dendrites identified as a monostratified small-to-medium retinal ganglion cell. (F) A bistratified retinal ganglion cell with EGFP expression after 2 days in culture. It was identified as a bistratified retinal ganglion cell. (Fi) A projected image of both layers of the bistratified retinal ganglion cell, with (Fii) an ON layer, and (Fiii) an OFF layer. Scale bar, 50 µm.

One of the advantages of our organotypic tissue culture of the adult rodent retina was the ability to carry it out in combination with variable gene transfer by particle-mediated acute gene transfer. We first examined EGFP transduction in retinal ganglion cells in rat retinas using particle-mediated acute gene transfer in vitro; EGFP was expressed as early as day 1 in culture (Figure 5A). After one day in culture, whole dendritic arbors were identified by EGFP expression in 50 to 300 cells per retina (Figure S2). The expression was rapid, occurring as early as one day after culturing. Even after 4-day culture, EGFP-expressing rat retinal ganglion cells seemed to be intact morphologically (Figure 5B). In the mouse retina, EGFP-expressing cells appeared as early as day 1 in culture (Figure 5C). After 4-day culture of adult mouse retina, we were able to observe EGFP expression in morphologically intact retinal ganglion cells (Figure 5D), more than 50 to 100 cells per retina.

We were able to identify subtypes of retinal ganglion cells as early as day 1 of culturing by their morphologies and EGFP distribution along the whole dendritic arbor. For example, Figures 5E and 5F show two typical subtypes of retinal ganglion cells. Figure 5E shows a monostratified small-to-medium retinal ganglion cell, which was identified as RG_B2_ by Sun et al [9]. Figure 5F shows a bistratified medium retinal ganglion cell, which was also identified as RG_D2_ by Sun et al [9]. This cell looked quite similar in morphology to On-Off directionally selective ganglion cells of rabbits and mice [10]–[12].

### Polarized transfer of fusion proteins into appropriate intracellular locations in retinal ganglion cells

To achieve induction of variable transgenes into adult rodent retina and to evaluate polarized transfer of fusion proteins into appropriate locations in retinal neurons after culture, we examined *PSD95-GFP* gene transfer of a postsynapse-targeting protein and newly-synthesized transgenic melanopsin (human *OPN4*, *hOPN4*) gene transfer as a membrane-targeting protein.

**Figure 6:**
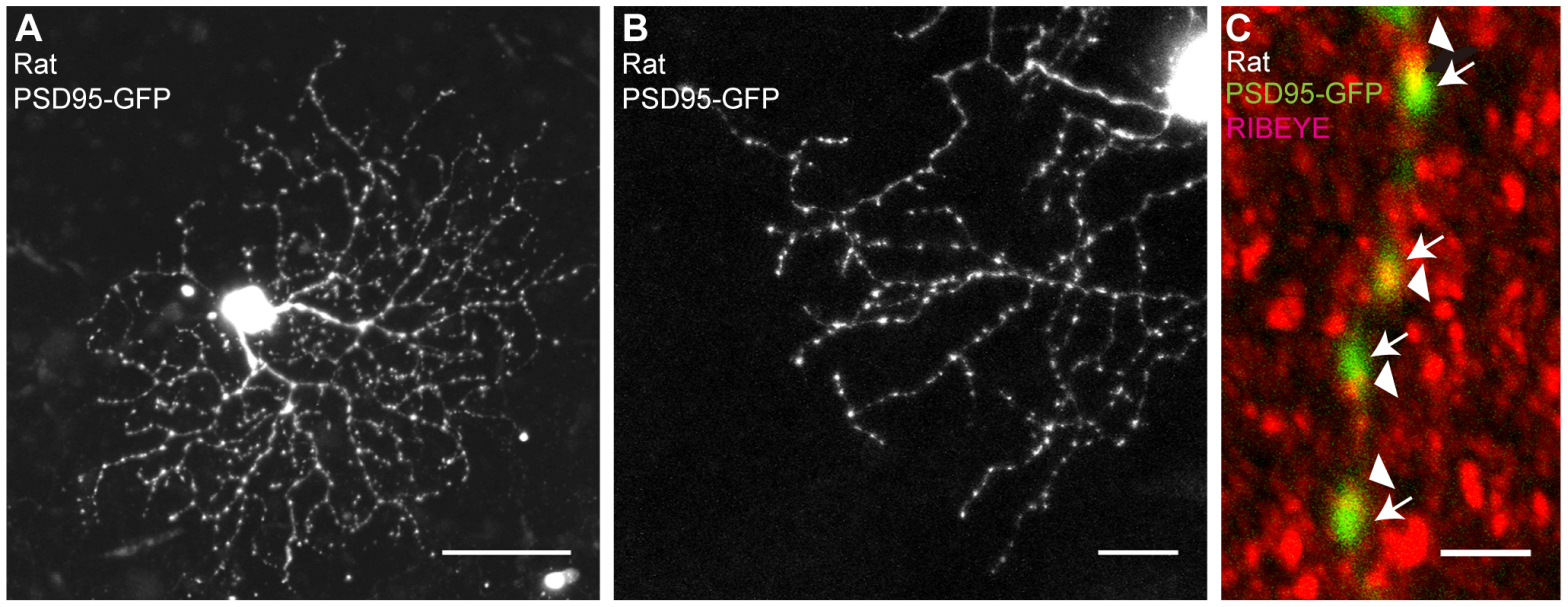
Transfection of *PSD95-GFP* and its distribution in rat retinal ganglion cells. (A) A rat retinal ganglion cell transfected with *PSD95-GFP* after 3 days in culture. Scale bar, 50 µm. (B) Highly magnified image of a part of the dendritic arbor of a rat retinal ganglion cell transfected with *PSD95-GFP* after 2 days in culture. Scale bar, 20 µm. (C) High-power view of dendrite of a rat retinal ganglion cell transfected with PSD95-GFP (green) and counterstained with an antibody against the synaptic ribbon component RIBEYE (CtBP2, red). A 2-µm-depth merged z-stack image is shown. Arrows and arrowheads indicate PSD95-GFP-labeled postsynaptic components and RIBEYE-labeled presynaptic components, respectively. Scale bar, 2 µm.

PSD95-GFP is a fusion protein of the synaptic marker postsynaptic density component PSD95 with green fluorescent protein (GFP) [13]. Most of the excitatory input synapses to ganglion cells from bipolar cells are mediated by aminomethylphosphonic acid (AMPA)-type glutamate receptors that have proximity relationships with PSD95 in their dendrites [14]; the puncta of PSD95-GFP should be localized at synaptic sites. As shown in Figures 6A and 6B, the PSD95-GFP puncta were distributed among dendritic arbors of retinal ganglion cells. Immunohistochemical study showed that PSD95-GFP puncta on dendrites were observed in close proximity to RIBEYE (CtBP2), one of proteins associated with the pre-synaptic ribbon complex (Figure 6C) [4].

**Figure 7:**
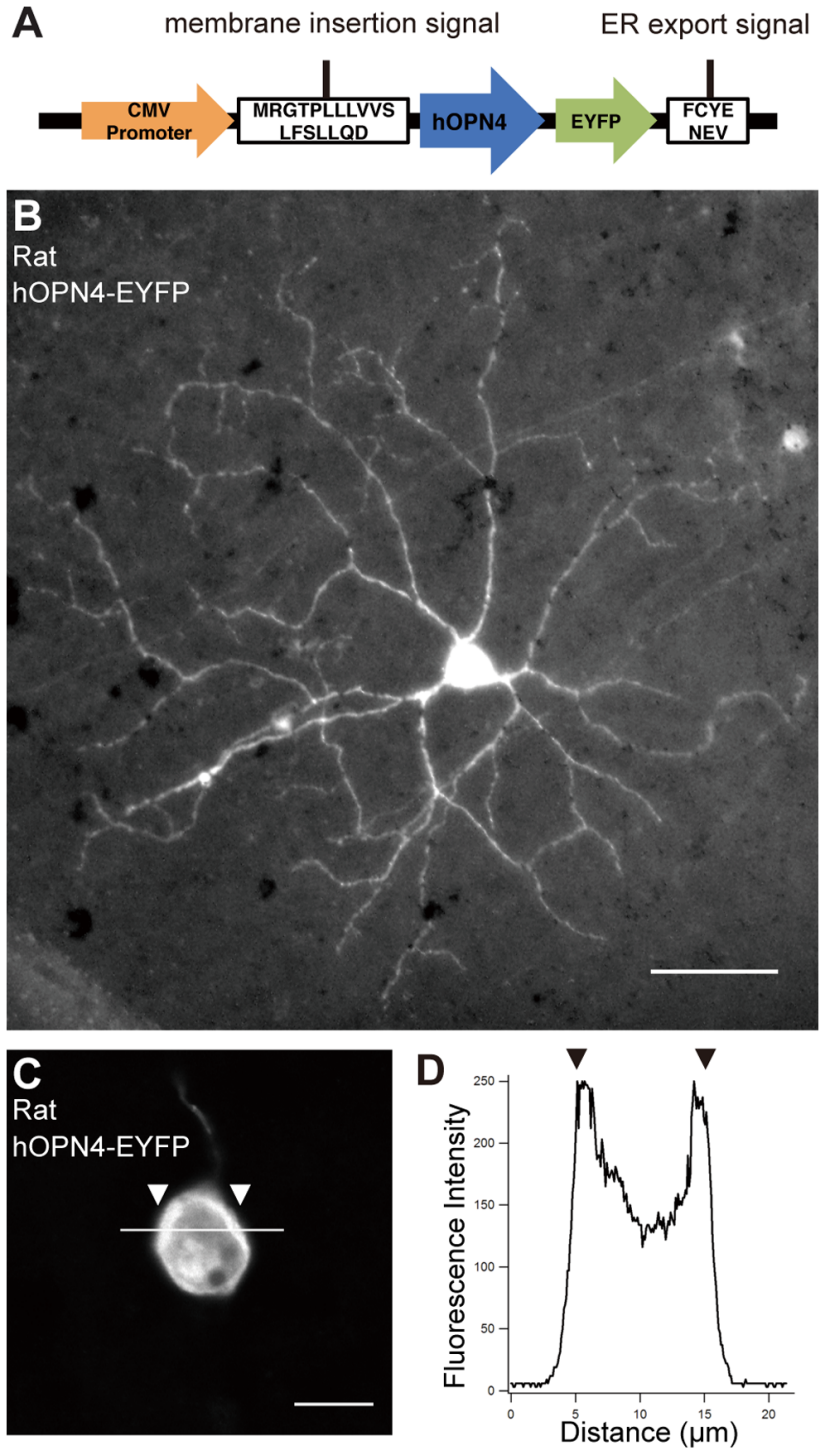
Membrane-targeting hOPN4-EYFP expression in rat retinal ganglion cells. (A) Primary structure of the EYFP-tagged hOPN4 construct with membrane-targeting signals of the N-terminal signal peptide derived from nAChR and the C-terminal ER export signal derived from Kir2.1. Expression was driven by the CMV promoter. (B) An example of rat retinal ganglion cells transfected with an expression plasmid for *hOPN4-EYFP* after 2 days in culture. The fluorescence in the cell body was saturated in this picture. Scale bar, 50 µm. (C) Highly-magnified view around the cell body of a *hOPN4-EYFP-*transfected rat retinal ganglion cell. Scale bar, 10 µm. (D) Fluorescence intensity along the bar on the cell body (0 to 255) indicates the membrane localization of hOPN4-EYFP. The cell membrane shows higher intensity at the arrowheads.

We also transfected a newly synthesized *hOPN4-EFYP* transgene (Figure 7). Melanopsin (OPN4) is a biomolecular photosensitive sensor that is a 7-transmembrane G-protein coupled receptor (GPCR) protein [15]–[17]. Wild-type melanopsin itself is normally inserted into membrane, but some transgenic melanopsin cannot be transferred into the membrane and accumulates in the endoplasmic reticulum (ER). To avoid this problem, we synthesized the transgenic *EYFP*-tagged *hOPN4* expression vector with membrane-targeting signals [18] (see Methods; Figure 7A). We transfected this plasmid into retinal ganglion cells by particle-mediated acute gene transfer. After 1- or 2-day culturing, we detected (by immunocytochemsitry) ganglion cells with EYFP. In Figures 7B and 7C, EYFP was distributed in the cell body as well as through the entire dendritic arbor in the transfected retinal ganglion cells. Accumulation of fluorescent signals in the cell surface (arrowheads in Figures 7C and 7D) indicated the membranous expression of this fusion protein.

We conclude that organotypic tissue culture of adult rodent retina permits acute variable gene manipulation by using particle-mediated acute gene transfer.

## Discussion

Over the last several years, the rodent retina has become a useful bioresource for retinal and ophthalmological research. However, in vitro culture systems using adult rodent retinas and variable gene transfer have yet to be established. Several researchers have described culture systems that used postnatal rodent retinas [19]–[22], but they were limited to certain purposes in biochemical and immunohistochemical assay of apoptotic cell death, glutamate toxicity, or circadian rhythms. For retinal neurophysiology in combination with molecular biological techniques, Koizumi et al [1] previously established an organotypic tissue culture system using adult rabbit retinas, and this organotypic culture method has been applied to several physiological retinal research projects [3], [4]. Recently, retinal neurophysiologists have become eager to share the advantages of the adult rodent retina as an animal model with flexible gene manipulation. Here we report a newly established organotypic tissue culture of the adult rodent retina (P35 – P56) optimized from Koizumi et al's adult rabbit retinal culture system [1].

In the present study, acute gene transfer was conducted by particle-mediated, ballistic transfer into retina [1], [23], but another option might be the use of an electroporation technique [24], [25]. In the preliminary study, only Mueller glial cells were transfected by electroporation in our in vitro culture system (data not shown), although further optimization must be examined. Virus-vector mediated gene transfer in vitro could be another option [26].

Using particle-mediated acute gene transfer, we have successfully transfected variable transgenes into individual retinal neurons, including several subtypes of retinal ganglion cells. The advantage of this system using the adult rodent retina was thought to be the rapidity and efficiency of acute gene expression. Gene expression after transfection occurred less than 24 hours after transfer, more rapid than the 48 hours needed for the organotypic culture system of adult rabbit retinas, probably because of the differences in promoter activity (such as the CMV promoter) in these species. This rapid expression of transgenes in adult rodent retinal tissue is one of advantages for the acute gene expression assay in our organotypic tissue culture of the adult rodent retina.

In our organotypic tissue culture of the adult rat retina, EGFP expression was stable and we always observed 50 to 300 morphologically intact cells with EGFP expression per rat retina (see Figures 5A, 5B, and S2). In mouse retina, more than 50 to 100 cells/retina seemed morphologically intact, but 17.3% of the mouse retinal tissue after organotypic culture showed less viability (Figure 3E, YO-PRO-1 staining). EGFP-expressing cells in these damaged areas showed abnormal morphology, including pathological swellings and thick dendrites (data not shown). These pathological morphologies were observed more often in mouse retinas after culture, and it was not clear what caused these changes. Further optimization for adult mouse retinas might be required. In other words, the morphology of the cells after the culture is a good indication for “healthiness” of the cultured retina. In conclusion, after the organotypic culture of adult mouse retina, it may be necessary to examine the morphology of the cells more carefully.

Electrophysiologically, light responses became less reliable after culture, probably because of loss of retinal pigmental epithelium and shorter outer segments of photoreceptors. However, we can emphasize that all of the rat retinal ganglion cells after 4-day culture were able to generate action potential by patch-clamp recordings and almost half of the rat retinas after culture showed light responses by trans-retinal field potential recordings (Figure 4). In particular, positive potentials were detected clearly in proportion to light intensity by trans-retinal field potential recordings. The positive potentials were thought to be light responses similar to b-wave in electroretinogram. In conclusion, it is difficult to conduct the experiments which require repetitive light exposure such as studies on visual information processing. If we could activate photocycles of photopigments by supplementing enzymes in the culture medium, we should have more reliable light responses after culture. Further investigation will be required.

Overall, this organotypic culture method is largely applicable to rat retinas, but it can be used for gene transduction studies in the retinas of both mice and rats. Further application of our organotypic culture system of adult rodent retinas is expected, especially for bioassays in combination with molecular biological approaches on individual cells of retinal neurons in adult rat and mouse retinas. Rat and mouse retinal neurons are divided into many subtypes [9]–[11], [27]–[31]; the neural circuits are quite complicated, because many subtypes of neurons are intricately intertwined with each other to process visual information [32]. To dissect neural circuits of the retina, molecular biological approaches such as RNA interference (RNAi) became more important in combination with electrophysiological, optogenical, biochemical, and immunohisotochemical studies. Our newly-established organotypic culture system for adult rodent retina is quite flexible for these purposes.

## Methods

Handling and euthanasia of animals were done in accordance with the guidelines provided by the National Institutes of Health Guide for the Care and Use of Laboratory Animals, and the experimental protocols were approved by the Institutional Animal Care and Use Committee of the National Institutes of Natural Sciences and the National Institute for Physiological Sciences (No. 09A241, No. 10A017, and No. 10A032).

### Isolation of rodent retinas

Male Wistar rats 5 to 8 weeks of age (P35 – P56) (Japan SLC, Hamamatsu, Japan), 129/SvEv mice (P49 – P56 age) (Taconic, Hudson, NY), and male C57BL/6J Jms mice (P35–P56) (Japan SLC) were used for culture and transfection experiments. Rats and mice were deeply anesthetized using halothane and euthanized with pentobarbital. The eyes were removed and immediately transferred to oxygenized Ames' medium (Sigma–Aldrich, St. Louis, MO) containing 0.192% sodium bicarbonate prior to hemisection. The retinas were teased off the sclera using fine forceps.

### Interphase chamber for organotypic tissue culture of adult rodent retinas

Adult rodent retinal culture was performed with modifications to the published protocol for the interphase culture system of adult rabbit retina [1], [2]. Rat and mouse retinas were placed ganglion cell side up on a 0.4-µm Millicell tissue culture insert (Millipore, Billerica, MA) and gentle suction was applied to the tissue for attachment to the membrane. Filter stands (3-cm diameter, 1.2-cm high) were cut from Delrin tubing, so that the Millicell filter rested on stands when it was placed into a 60-mm diameter x 20-mm depth cell culture dish (“deep dish”; Nunc, Rochester, NY) (Figures 1A and 1B). More than 26 mL Ames' medium (Sigma–Aldrich, St Louis, MO) per retina (containing 0.192% sodium bicarbonate, 100 U/mL penicillin, 100 µg/mL streptomycin, and 0.292 mg/mL L-glutamine [Invitrogen, Carlsbad, CA]) was added to the dish and was supplemented with 10% horse serum (Sigma–Aldrich), with the retina contacting the medium via the Millicell filter on the photoreceptor side and the incubator atmosphere (5% CO_2_, 37°C, humidified) over the ganglion cell side. All further manipulations, including particle-mediated acute gene transfer, were carried out with the retina attached to the filter. During culture in the CO_2_ incubator, the medium was agitated constantly at 55 rpm using an orbital shaker (MIR-S100C; SANYO, Tokyo, Japan) and was exchanged daily.

### Cell viability assay after culture

Cell viability was assessed by using a Vybrant apoptosis assay kit (Invitrogen). Briefly, rat and mouse retinas after 4 days of culture were incubated with 100 nM YO-PRO-1 for 1 hour. Acutely isolated retinas were also stained within 1–2 hours after isolation as controls. YO-PRO-1 positive cells were clustered so that we defined a clustered area as YO-PRO-1 positive area, because these fluorescents were distinct from background. We measured the area against the whole retinal area using ImageJ (National Institutes of Health, Bethesda, MD).

### Electrophysiological recordings from cultured retinas

For all electrophysiological recordings, retinas of dark-adapted animals were prepared under faint red light and were cultured under dark.

#### 1. Patch-clamp recordings

For patch-clamp recordings, the recording techniques were conventional and described previously [33]. Briefly, patch pipettes (approximately 10 MΩ resistance) were pulled from Pyrex tubing on a micropipette puller (P-97; Sutter Instrument, Novato, CA). For whole-cell patch-clamp recordings, the pipette solution consisted of 125 mM K-gluconate, 5 mM KCl, 10 mM Hepes, 1 mM CaCl_2_, 1 mM MgCl_2_, and 11 mM EGTA (pH adjusted to 7.2 with KOH). The piece of retina was placed in a recording chamber, ganglion cell layer up, and continuously perfused at a rate of 1 to 2 mL/min with oxygenated extracellular solution (containing 125 mM NaCl, 2.5 mM KCl, 2 mM CaCl_2_, 1 mM MgCl_2_, 26 mM NaHCO_3_, 1.25 mM KH_2_PO_4_, and 12 mM glucose. The extracellular solution was continuously oxygenated with 5% CO_2_/95% O_2_ and kept between 32 and 35°C. For loose cell-attached patch-clamp recordings, the pipette solution was filled with the extracellular solution. The recording pipette was connected to the input stage of a patch-clamp amplifier Axopatch 200B (Axon Instruments, Foster City, CA), and signals were sampled at 10 kHz with DigiData 1322A interface-type and pCLAMP8 software (Axon Instruments). The liquid junction potential was measured as 11 mV (Vm = Vp−11 mV) and corrected after recordings. Subsequent analysis was done by custom-made procedures in Igor Pro (WaveMetrics, Lake Oswego, OR). For light exposure, white light was applied thorough an objective lens with an epi-illumination system from a mercury lamp. The intensity of the light was measured by using a photometer (LS-100; Konica Minolta, Tokyo, Japan) or an illuminance meter (T-10M; Konica Minolta).

#### 2. Trans-retinal field potential recordings

In this study, we also applied trans-retinal field potential recordings, an imitation of in vitro electroretinogram, which represented the sum of all electrical activity of retinal neurons following exposure to a light stimulus [8] (Figure S1). The cultured retina (in a culture dish) was mounted on a custom-made, electrically-insulated holder. In the holder, the retina was placed on a Millicell filter with photoreceptor-side down. During recordings, the whole device was placed in a CO_2_ incubator, at 37°C, under darkness. Light stimulus was supplied by a LED (white) from the bottom (photoreceptor side). Maximum luminance at the surface of the retina was 450 cd/m^2^. Timing and intensity of light stimulus were controlled by a function generator (WF1973; NF Corporation, Yokohama, Japan). A reference Ag/AgCl electrode was placed in the medium on the retinal ganglion cell side, and a recording Ag/AgCl electrode was placed in the medium of the culture dish. Electrical signals were amplified with a differential amplifier (MEG-5100; Nihon Kohden, Tokyo, Japan) and signals were digitally sampled with a digital phosphor oscilloscope (TDS 3014B; Tektronix, Beaverton, OR) thorough high-cut and low-cut filter settings of 0.5 and 300 Hz, respectively, Every light stimulus was exposed at an interval of 0.5 to 10 minutes.

### Particle-mediated acute gene transfer of plasmids

A Helios gene gun system (Bio-Rad, Hercules, CA) was used for particle-mediated acute gene transfer to retinal neurons (Figure 1C). The following expression vectors were used: *CMV-EGFP* (*pEGFP-N1*; Clontech, Palo Alto, CA), *CMV-PSD95 GFP* fusion [13] (gift from Dr. Fukata), and *CMV-human OPN4 EYFP* fusion. Membrane insertion signals and ER export signals were added to the N and C terminals of *hOPN4-EYFP*, respectively (Figure 7A) [18].

Plasmid-coated gold microcarriers (1.6-µm gold) were prepared as described in the manufacturer's protocol. To make particle-mediated acute gene transfer bullets, DNA-coated gold microcarriers (10 or 20 µg of DNA per 10 mg of gold) were suspended in 1.6 or 3.2 mL ethanol and loaded into Tefzel tubing (Bio-Rad) using their Tubing Prep Station (Bio-Rad). The particle-mediated acute gene transfer was held 5 mm above the tissue and the gold microcarriers coated with the appropriate plasmids were propelled into the retina at a delivery pressure of 110 psi.

### Immunohistochemistry, Nissl staining, and image acquisition

Harvested retinas were fixed in 4% paraformaldehyde in 0.1 M phosphate buffer or phosphate buffered saline at room temperature for 0.5 to 1 h. For vertical sections, each fixed retina was embedded in Tissue-Tek O.C.T. Compound (Sakura Finetechnical Co., Tokyo, Japan), frozen, and sectioned at 20-µm thickness using a Cryostat (Leica CM 3050; Leica Microsystems, Wetzlar, Germany). Primary antibodies against ChAT (1∶200, goat polyclonal, Millipore), GFP (1∶1000, rabbit polyclonal; Molecular Probes, Eugene, OR), GFP (1∶500, goat polyclonal; Rockland Immunochemicals, Gilbertsville, PA), and CtBP2 (RIBEYE) (2 µg/ml, rabbit polyclonal; BD Biosciences, San Jose, CA) were used. The primary antibody was visualized using Alexa Fluor 594, Alexa Fluor 488, and Alexa Fluor 555-conjugated secondary antibodies (Molecular Probes). Vertical sections were counterstained with the Hoechst 33342 nuclear dye (Sigma–Aldrich). Samples were mounted and coverslipped using Fluoromount-G (SouthernBiotech, Birmingham, AL). For Nissl staining, the sections were treated with 1% cresyl violet acetate for 15 min. Images were captured using a fluorescence microscope (BX50; Olympus, Tokyo, Japan) equipped with CCD camera (DP72; Olympus), fluorescence microscopy (BZ-9000K; KEYENCE, Osaka, Japan), fluorescence stereo microscope (MZ10F; Leica Microsystems) equipped with digital color camera (EC3; Leica Microsystems) or confocal laser scanning microscopy (LSM510; Carl Zeiss, Jena, Germany). The resulting images were adjusted for brightness and contrast using ImageJ (National Institutes of Health) or Photoshop CS (Adobe; San Jose, CA).

### Statistical evaluation

The spacing of cell bodies of starburst amacrine cells in the inner nuclear layer (OFF layer) and the ganglion cell layer (ON layer) was analyzed using the Density Recovery Profile (DRP) method, calculated in MatLab (The Mathworks, Lowell, MA) [34]. The concept of DRP analysis used the spatial autocorrelogram, as described previously [35].

## Supporting Information

Figure S1 Trans-retinal field potential recording of acutely isolated rat retina elicited by light stimulus. (A) Schematic drawing of the equipment for trans-retinal field potential recordings. The cultured retina was mounted on electrically insulated holder with a photoreceptor side down. Light stimulus was irradiated by LED (white). Maximum luminance was 450 cd/m^2^ at the surface of retina. During recordings, the equipment was placed in CO_2_ incubator, 37°C, under darkness. (B) An example of light-elicited potential changes from acutely isolated rat retina. Light responses were recorded in proportion to the intensity of light stimulus. Light-elicited negative potentials (arrow heads) followed by positive potentials (arrows) were detected.
(0.37 MB TIF)

Figure S2 Expression of fluorescent protein observed in whole-tissue rat retina after culture. Low-power micrograph of adult rat retina transfected with an expression plasmid for hOPN4-EYFP after 2 days in culture. More than 286 cells were expressing EYFP in this retina. Scale bar, 1 mm.
(0.81 MB TIF)
