# Supplementary figures and images for: Organotypic Tissue Culture of Adult Rodent Retina Followed by Particle-Mediated Acute Gene Transfer In Vitro
Source: PLoS One. 2010 Sep 23;5(9):e12917. doi: 10.1371/journal.pone.0012917 (PMC2944845; doi:10.1371/journal.pone.0012917)

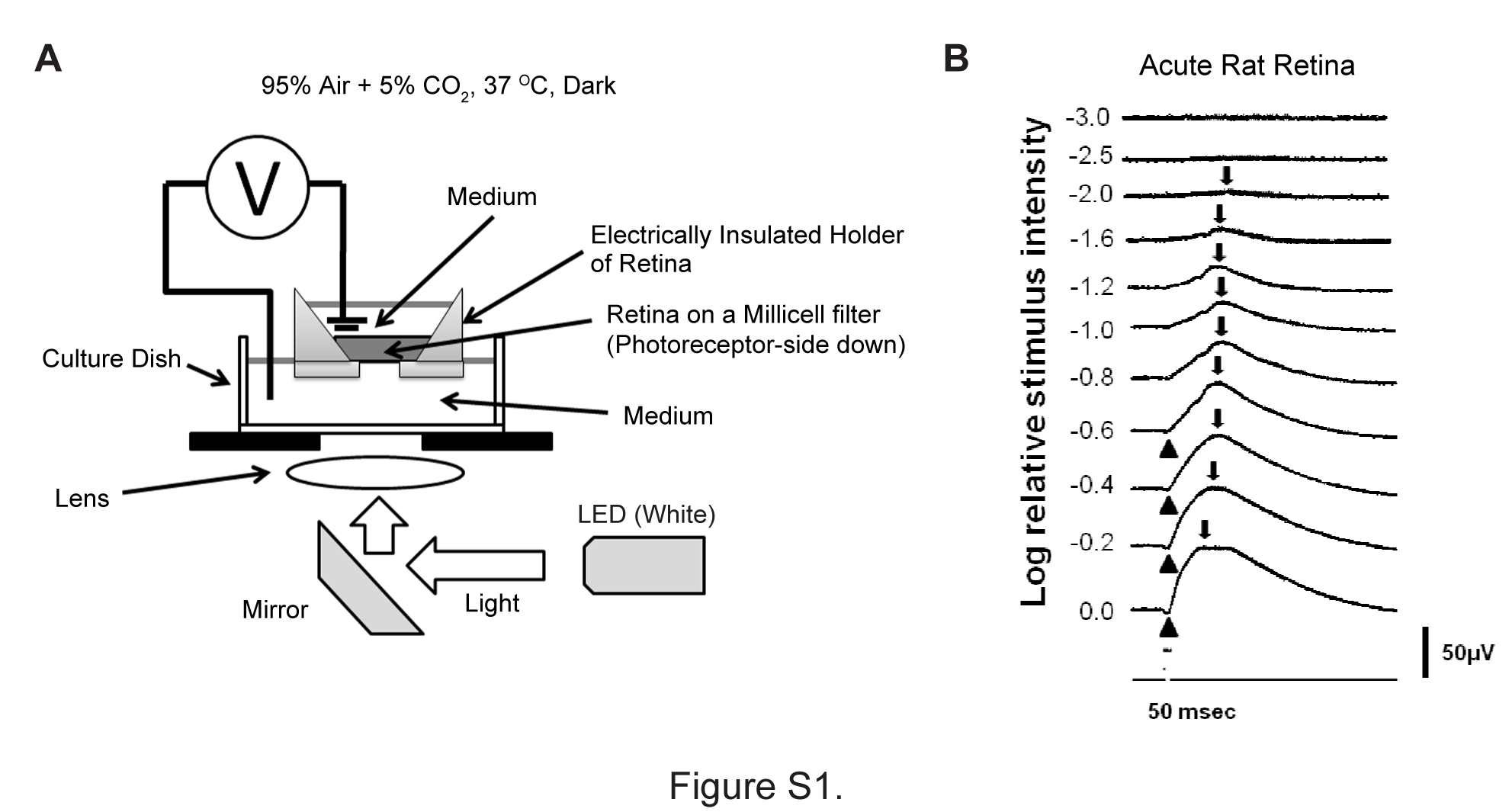

Supplement: Figure S1 — Trans-retinal field potential recording of acutely isolated rat retina elicited by light stimulus. (A) Schematic drawing of the equipment for trans-retinal field potential recordings. The cultured retina was mounted on electrically insulated holder with a photoreceptor side down. Light stimulus was irradiated by LED (white). Maximum luminance was 450 cd/m2 at the surface of retina. During recordings, the equipment was placed in CO2 incubator, 37°C, under darkness. (B) An example of light-elicited potential changes from acutely isolated rat retina. Light responses were recorded in proportion to the intensity of light stimulus. Light-elicited negative potentials (arrow heads) followed by positive potentials (arrows) were detected. (0.37 MB TIF) [file pone.0012917.s001.tif]

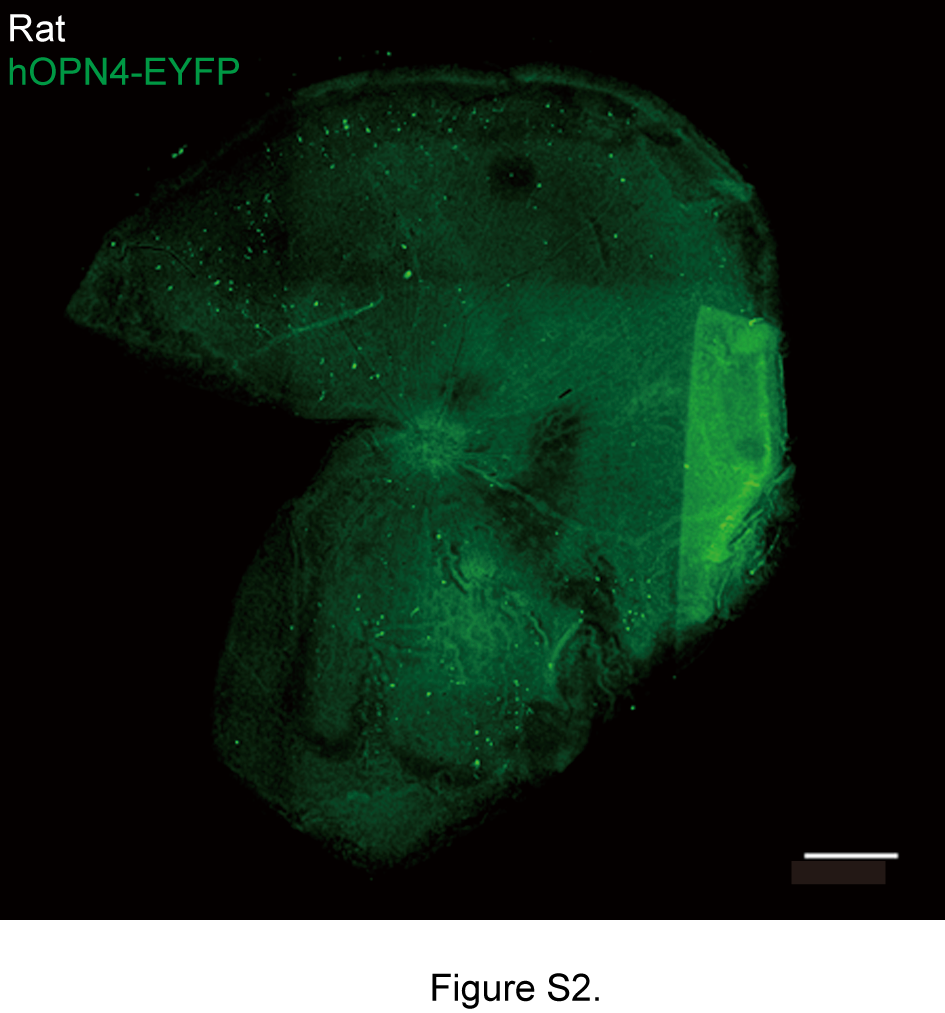

Supplement: Figure S2 — Expression of fluorescent protein observed in whole-tissue rat retina after culture. Low-power micrograph of adult rat retina transfected with an expression plasmid for hOPN4-EYFP after 2 days in culture. More than 286 cells were expressing EYFP in this retina. Scale bar, 1 mm. (0.81 MB TIF) [file pone.0012917.s002.tif]
